# Regression Analysis of the Dielectric and Morphological Properties for Porous Nanohydroxyapatite/Starch Composites: A Correlative Study

**DOI:** 10.3390/ijms23105695

**Published:** 2022-05-19

**Authors:** Chong You Beh, Ee Meng Cheng, Nashrul Fazli Mohd Nasir, Mohd Shukry Abdul Majid, Shing Fhan Khor, Mohd Ridzuan Mohd Jamir, Emma Ziezie Mohd Tarmizi, Kim Yee Lee

**Affiliations:** 1 Faculty of Electronic Engineering Technology, Universiti Malaysia Perlis (UniMAP), Arau 02600, Perlis, Malaysia; neilbeh@hotmail.com (C.Y.B.); nashrul@unimap.edu.my (N.F.M.N.); 2 Advanced Communication Engineering (ACE) Centre of Excellence, Universiti Malaysia Perlis (UniMAP), Arau 02600, Perlis, Malaysia; 3 Faculty of Mechanical Engineering Technology, Universiti Malaysia Perlis (UniMAP), Arau 02600, Perlis, Malaysia; shukry@unimap.edu.my (M.S.A.M.); ridzuanjamir@unimap.edu.my (M.R.M.J.); 4 Faculty of Electrical Engineering Technology, Universiti Malaysia Perlis (UniMAP), Arau 02600, Perlis, Malaysia; sfkhor@unimap.edu.my; 5 Centre of Foundation Studies for Agricultural Science, Universiti Putra Malaysia, Serdang 43400, Selangor, Malaysia; emma@upm.edu.my; 6 Lee Kong Chian Faculty of Engineering & Science, Sungai Long Campus, Tunku Abdul Rahman University, Jalan Sungai Long, Sungai Long City, Cheras, Kajang 43000, Selangor, Malaysia; kylee@utar.edu.my

**Keywords:** porous composite, microstructural features, dielectric properties, starch, hydroxyapatite nanoparticle

## Abstract

This paper aims to investigate the dielectric properties, i.e., dielectric constant (ε′), dielectric loss factor (ε″), dielectric tangent loss (tan δ), electrical conductivity (σ), and penetration depth (D_p_), of the porous nanohydroxyapatite/starch composites in the function of starch proportion, pore size, and porosity over a broad band frequency range of 5 MHz–12 GHz. The porous nanohydroxyapatite/starch composites were fabricated using different starch proportions ranging from 30 to 90 wt%. The results reveal that the dielectric properties and the microstructural features of the porous nanohydroxyapatite/starch composites can be enhanced by the increment in the starch proportion. Nevertheless, the composite with 80 wt% of starch proportion exhibit low dielectric properties (ε′, ε″, tan δ, and σ) and a high penetration depth because of its highly interconnected porous microstructures. The dielectric properties of the porous nanohydroxyapatite/starch composites are highly dependent on starch proportion, average pore size, and porosity. The regression models are developed to express the dielectric properties of the porous nanohydroxyapatite/starch composites (R^2^ > 0.96) in the function of starch proportion, pore size, and porosity from 1 to 11 GHz. This dielectric study can facilitate the assessment of bone scaffold design in bone tissue engineering applications.

## 1. Introduction

In tissue engineering, numerous efforts have been made in research to find alternative materials with similar microstructural features as a natural bone for bone defect reconstruction [1,2]. The microstructural features (pore size, pore distribution, porosity, and pore interconnectivity) of a bone scaffold are the main factors that lead to biological activities (cell adhesion, cell migration, cell proliferation, and cell differentiation) during bone regeneration [3,4]. Several characterization techniques (microscopy, micro-computed tomography, density determination, and ultrasonic testing) are available for evaluating bone scaffold microstructures, but most of the techniques are destructive, costly, and time-consuming. Non-destructive techniques have been extensively studied to explore the potential of an alternative characterization for the microstructural characterization of bone scaffolds [5,6].

Hydroxyapatite (Ca_10_(PO_4_)_6_(OH)_2_), a bioceramic, shows excellent biodegradability, bioactivity, biocompatibility, and osteoconductivity. Consequently, it is versatile in diverse biomedical applications, e.g., gene therapy, regenerative medicine, drug delivery, and tissue/implant engineering [3,7]. Hydroxyapatite is commonly used as an artificial bone substitute because of its physical, chemical, and biological similarities with the natural bone. Hydroxyapatite has surface charges and electrical polarizability because of its dielectric behavior, which is beneficial for biocompatibility enhancement and tissue regeneration [8,9,10]. Nonetheless, the brittle nature of hydroxyapatite is a very critical limitation in biomedical applications. Thus, a polymer is applied to hydroxyapatite to form composites [3,4]. Normally, polymeric materials are easy to be processed, inexpensive, accessible, and adhesive [11,12]. Starch is one of the most abundant and renewable polymeric materials in the world. Starch is used in a wide variety of biomedical applications due to its notable physical, chemical, and biological properties [11,13]. Recently, nanosized hydroxyapatite has been extensively used in biomedical applications because of its high surface area, which is efficient in the numerical study of the cell activities and local growth factors enhancement [9,14]. The application of the nanosized hydroxyapatite in polymer matrices enhances the microstructure and polarizability to fulfill the requirements in biomedical applications [15,16].

Radiofrequency electromagnetic radiation can be used in material characterization. The dielectric properties measurement is useful in determining the quality assessment of a material, especially composite material characterization [13,17,18]. Bulk density, composition, microstructure, porosity, filler volume fraction, filler shape and size, filler orientation, material phase properties, and material interfacial interaction are the functions of dielectric properties [12,19,20]. When a composite is subjected to an oscillated electric field (time-varying field or electromagnetic wave), the electromagnetic wave propagation mechanism, i.e., absorption, transmission, reflection, and scattering, induces displacement currents and conduction currents, which leads to polarization and energy dissipation, respectively [21,22]. Hierarchical porous structures and heterogeneous architecture of the bioceramic/polymer composite are important in order to improve the dielectric response, which contributes to polarization and energy dissipation [7,23,24]. In dielectric measurements, the operating frequency causes variation in the dielectric responses of every composite since different polarization mechanisms contribute to the composite’s overall response to an applied electric field with various frequencies [13,18]. The material composition, microstructure feature, and surface morphology of the bone scaffold play an extremely important role in the bone healing process. These parameters are responsible for the biological activity of the bone scaffold during bone tissue regeneration [1,3]. Hence, a rapid and effective characterization is required for these parameter evaluations in order to optimize the bone scaffold quality. Presently, less research regarding the effect of the microstructural features on the dielectric behaviors of the bioceramic-based bone scaffold with various polymer proportions was reported. The dielectric properties of natural bone are influenced by its matrix composition and pore density. The bone scaffold should exhibit low and stable dielectric responses due to the presence of air-filling voids. Air is an excellent dielectric medium. The dielectric responses of bone scaffold mainly depend on the number of air-filling voids in the matrix. The bone scaffold with high porosity results in low dielectric responses, which is favorable for bone cell growth and proliferation [4,25]. The dielectric analysis of the porous bioceramic/polymer composites over 5 MHz–12 GHz have not been reported extensively in research publication. Hence, this will bring about a much-needed better understanding of the relationship between microstructural features, material proportions, and dielectric behaviors over the 5 MHz–12 GHz frequency range in biomedical applications.

In this study, a dielectric study is conducted on the porous bioceramic/polymer composites as a function of polymer proportion over a broad band frequency range of 5 MHz–12 GHz. In the meantime, the influence of material proportion and the microstructure features on the associated dielectric properties is investigated too. Hydroxyapatite nanoparticles (nHA) and starch (S) are used as the bioceramic and polymer components of the porous composite, respectively. The porous nHA/S composites were prepared using 30, 40, 50, 60, 70, 80, and 90 wt% of the starch proportion. The dielectric properties of the porous composites elucidate dielectric constant (ε′), dielectric loss factor (ε″), dielectric tangent loss (tan δ), electrical conductivity (σ), and penetration depth (D_p_). ε′ and ε″ define the material’s storage and dissipate capability of electromagnetic energy, respectively [24,26]. tan δ and σ represent the material’s capability to attenuate electromagnetic energy and pass electrical current, respectively [27,28]. D_p_ indicates the depth of media that electromagnetic waves can penetrate [11]. The effect of microstructural features and incorporated material on the dielectric properties of the porous composites is important in the development of a nondestructive sensing system. It is associated with quality assessments during bone scaffold design and fabrication.

## 2. Results and Discussion

### 2.1. Morphological Analysis

**Figure 1:**
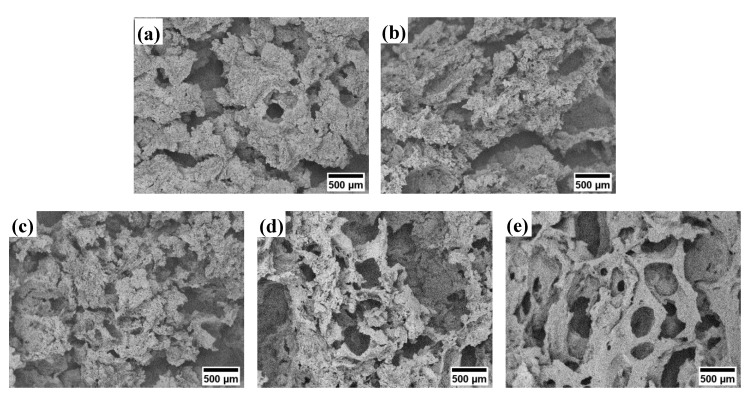

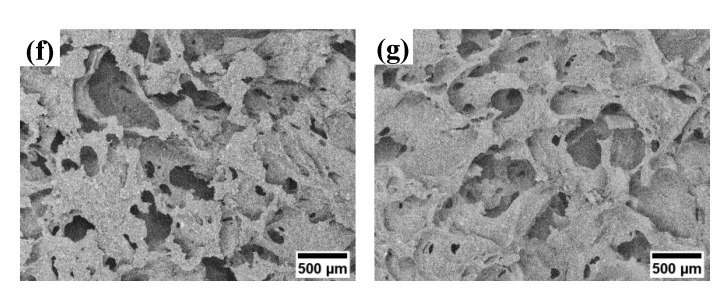
SEM images of the porous nHA/S composites containing (**a**) 30 wt% (H7S3), (**b**) 40 wt% (H6S4), (**c**) 50 wt% (H5S5), (**d**) 60 wt% (H4S6), (**e**) 70 wt% (H3S7), (**f**) 80 wt% (H2S8), and (**g**) 90 wt% (H1S9) of starch proportion.

**Figure 2:**
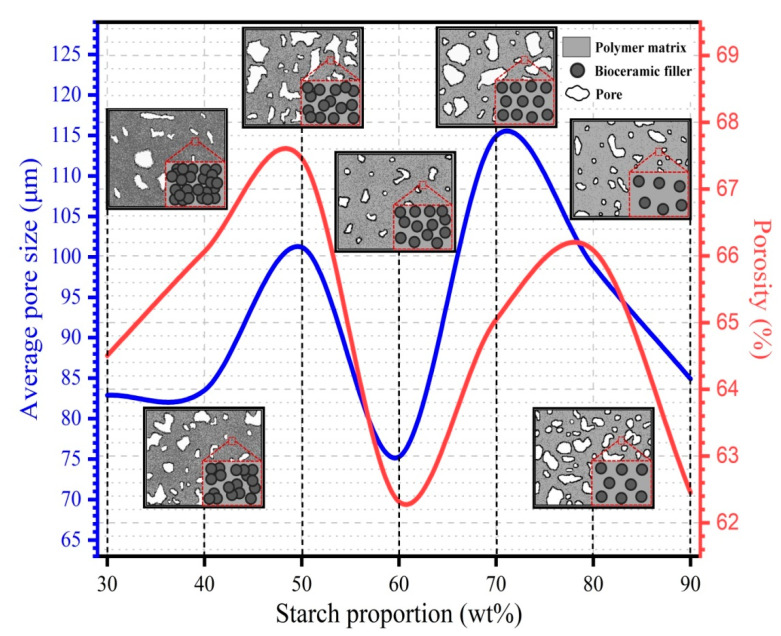
The effect of starch proportion on average pore size and porosity of the porous nHA/S composites.

The SEM images depict the microstructure of the porous nHA/S composites, as shown in Figure 1. The starch proportion of the porous nHA/S composites influences the morphological feature formation significantly. Figure 2 shows the effect of starch proportion on the average pore size and porosity of the porous nHA/S composites. Large irregular cluster formation can be observed in Figure 1a, which is indicated by the porous nHA/S composite with the 30 wt% starch proportion. This is attributed to the extremely saturated proportion of the hydroxyapatite nanoparticles. The separation between the hydroxyapatite nanoparticles is closer when the proportion of nanoparticles is high. It induces the formation of the clusters that are due to the significant agglomeration effect. It is not conducive to the formation of porous structures [14,29,30,31]. It causes a decrement in average pore size (82.9 μm) and porosity (64.5%) of the H7S3 composite, as shown in Figure 2.

It can be seen that in the porous nHA/S composites (Figure 2), the average pore size and porosity increase when the starch proportion increases from 30 wt% to 40 wt%. In the meantime, the average pore size and porosity of the porous nHA/S composite (Figure 2) are increased to 83.5 μm and 66.1%, respectively. The H6S4 composite exhibit smaller cluster structures than the other proportion. It indicates the presence of the micropores too within the composite matrices, as shown in Figure 1b, which contributes to the pore interconnectivity of the porous composite. The agglomeration effect of the hydroxyapatite nanoparticles is reduced because of the increment in the starch proportion, where the micropores of the porous nHA/S composite are formed by the interstices among the nonagglomerated composite matrices. In Figure 1c, the H5S5 composite with 50 wt% starch proportion displays the loose microstructure with a massive number of macropores and micropores. As shown in Figure 2, the average pore size (101.2 μm) and porosity (67.5%) increase as the starch proportion of the porous nHA/S composite increase from 40 wt% to 50 wt%. The H5S5 composite exhibits a higher porosity and larger average pore size as compared to the porous nHA/S composites with low starch proportion (30 wt% and 40 wt%). This might be due to their conversion of microstructures from closed pores to open pores, leading to higher interconnectivity [29,32,33].

Although the proportion of starch in porous nHA/S composite increases from 50 to 60 wt% and promotes the minimization of agglomerated hydroxyapatite nanoparticles, there is a decrement in average pore size (75.3 μm) and porosity (62.3%) of the H4S6 composite as shown in Figure 2. It might be due to severe inhomogeneous pore size distribution and the incorporation of pore occurring. If that is the case, H4S6 is unable to present effective interconnected porous structures and good agreement with the SEM image, as shown in Figure 1d. However, it can be observed that the further increment in starch proportion (from 60 to 70 wt%) in the porous nHA/S composites results in the increment in average pore size (114.9 μm) and porosity (65.0%), as shown in Figure 2. The porous structure of the H3S7 composite is rather regular and uniform in shape, as shown in Figure 1e. It is because sufficient starch proportion of the porous nHA/S composites enables full and complete incorporation of a starch matrix with the hydroxyapatite nanoparticles. It contributes to the formation of rigid porous structures. In Figure 1f, the H2S8 composite has a hierarchical porous microstructure that is attributed to the organized network of interconnected pores. As the starch proportion of the porous nHA/S composites increases from 70 to 80 wt%, the average pore size (98.9 μm) decreases. Meanwhile, the porosity (66.1%) increases, as shown in Figure 2. It is explicable through the uniform dispersion of the hydroxyapatite nanoparticles in the starch matrices. The relatively high starch proportion (80 wt%) facilitates the sufficient small pores to connect with the wall of larger pores to form macropores and micropores with high interconnectivity [32,34,35]. The SEM image of the H1S9 composite in Figure 1g shows that the micropore-dominant porous structure has numerous flat surfaces free from any pore features. The H1S9 composite has an average pore size of 84.9 μm and a porosity of 62.5%, as shown in Figure 2. It can be observed that when the starch proportion increases from 80 to 90 wt%, the average pore size and porosity of porous nHA/S composite decreases (Figure 2). This might be due to the porous nHA/S composite with the highest starch proportion, but insufficient hydroxyapatite nanoparticles result in the reduction in the pore wall rigidity. It provokes the collapse of the pore wall, thereby reducing the average pore size and the porosity.

### 2.2. Dielectric Analysis

#### 2.2.1. Dielectric Constant, ε′

**Figure 3:**
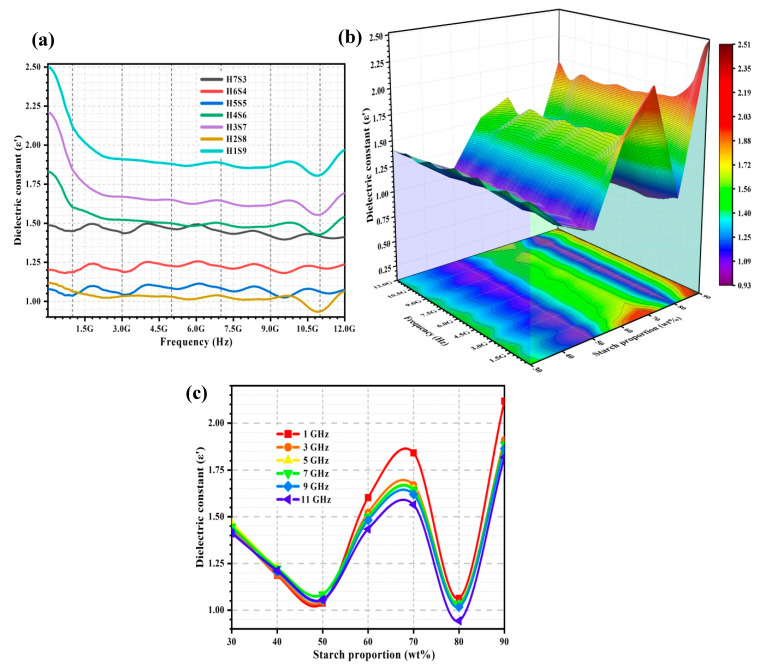
ε′ (**a**) of the porous nHA/S composites, 3D representation of ε′ of the porous nHA/S composites (**b**), and ε′ curves of the porous nHA/S composites at various selected frequencies (**c**).

Figure 3a shows the dielectric constant (ε′) spectra of the porous nHA/S composites. Figure 3b illustrates the relationship among ε′, starch proportion, and frequency within a range of 5 MHz to 12 GHz and starch proportion from 30 to 90 wt%. The variation of ε′ of the porous nHA/S composites at 1, 3, 5, 7, 9, and 11 GHz over starch proportion can be observed in Figure 3c. The ε′ of the porous nHA/S composites with various starch proportions exhibits a nonlinear decrement when the frequency increases. It is as shown in Figure 3a. The distinct discrepancies between the ε′ of the porous nHA/S composites might be due to the dependency of ε′ of porous composites on the microstructural features and the proportions of participating materials [32,36,37].

The ε′ of the porous nHA/S composites varies sinuously minor, as shown in Figure 3a, especially the porous composite with the lower starch proportion (H7S3, H6S4, and H5S5). These three porous composites have higher proportions of the hydroxyapatite nanoparticles (50–70 wt%). Significant interface reflections, space–charge accumulations, and polarized oxygen-containing functional groups of the hydroxyapatite nanoparticles are expected in the porous nHA/S composites under an applied electric field [13,38,39,40]. The ε′ of the porous nHA/S composites slowly decrease as the starch proportion increases from 30 to 50 wt%, as shown in Figure 3b,c. As the hydroxyapatite content decreases, the reduction in the hydroxyapatite nanoparticles’ permanent dipoles (phosphate and hydroxyl ions) leads to the decrement in ε′. The polarization mechanisms for the porous nHA/S composites with the higher hydroxyapatite content (> 50 wt%) are mainly dominated by the dipole–dipole interaction of the phosphate and hydroxyl ions when it is exposed to the applied electric field, especially the large clusters of agglomerated nanoparticles [9,12,26,41]. In microstructure, the average pore size and the porosity of the porous nHA/S composites increase with starch proportion from 30 to 50 wt% (Figure 2). The collective dipole polarization of the hydroxyapatite nanoparticles is suppressed [36,37,39].

It can be observed that the ε′ increases with the starch proportion of the porous nHA/S composites from 50 to 70 wt%, as shown in Figure 3b,c. This might be due to the proportion of the porous nHA/S composites reaching a critical range that leads to the formation of the large micro-capacitor network and strong interfacial or space–charge polarization (Maxwell–Wagner–Sillars effect), which can enhance the ε′. It is known as the dielectric percolation threshold effect [10,14,19,42]. The hydroxyapatite nanoparticles entrapped in the starch matrices, which are separated by air voids in the porous nHA/S composites, can be served as multiple micro-capacitor networks with a large capacity to store electric charges during polarization [7,10,43]. The nanosized hydroxyapatite particles dispersed in the porous nHA/S composite would contribute to the larger interfacial areas, which leads to substantial space–charge accumulation at the heterogeneous interfaces. It enhances the interface polarization [30,43,44,45]. The strong dielectric response (Figure 3b) and the higher ε′ (Figure 3c) of the porous nHA/S composite can be noticed at the 70 wt% starch proportion. It mainly occurs at a low frequency that the dielectric relaxation arises from the interfacial polarization. The largest average pore size of the H3S7 composite with high porosity (as shown in Figure 2) facilitates the multiple reflections/scattering of the electromagnetic waves in the porous microstructures of the composite. It extends the propagation path of the electromagnetic waves that lead to enhancement of the interfacial polarization [28,31,34,46].

The ε′ of the H2S8 composite is much lower than the other porous nHA/S composites, as shown in Figure 3a. Meanwhile, the decline in the ε′ of the porous nHA/S composites as the starch proportion increases from 70 to 80 wt% (as shown in Figure 3c) may be attributed to the significant adhesion between hydroxyapatite nanoparticles and polymeric matrices of starch. Abundant starch proportion in the porous nHA/S composites enables the hydroxyapatite nanoparticles to disperse evenly to restrict the mobility of the dipoles in the polymer chain of starch. Additionally, the hydroxyapatite nanoparticles are separated to suppress the space–charge polarization among the dipolar moieties of the nanoparticles under an alternating electric field. It is also known as the dielectric confinement effect [2,23,29,47]. Meanwhile, the H2S8 composite exhibits a higher porosity (Figure 2), which can lead to a significant decrement in the dielectric constant since air voids have a low dielectric constant (ε′ = 1) [44,47,48]. It can be seen from the comparison that the H1S9 composite with the highest starch proportion (lowest hydroxyapatite nanoparticle proportion) has the highest dielectric constant, as shown in Figure 3a,c. This should be attributed to the fact that the smaller average pore size and lower porosity of the H1S9 composite with a denser structure (Figure 2) are the dominant factors that determinate the relatively highest dielectric constant [33,49,50].

Regression analysis is conducted to develop regression equations. The function of ε′ of the porous nHA/S composites is the starch proportion (X_1_), pore size (X_2_), and porosity (X_3_). The relationship among parameters of ε′, X_1_, X_2_, and X_3_ in the porous nHA/S composites can be described by the developed regression models shown in Table 1. Their relationship at 1, 3, 5, 7, 9, and 11 GHz was analyzed. The regression models consist of a single independent variable and two independent variables. The positive sign of the coefficient demonstrates the synergistic effect, whereas the negative sign represents the antagonistic effect. The starch proportion and porosity have a positive effect on the dielectric constant of the porous nHA/S composites for all frequencies, as shown in Table 1. By contrast, the pore size has a negative effect on the dielectric constant of the porous nHA/S composites for all frequencies (Table 1). As shown in Table 1, the coefficient of determination (R^2^) was always above 0.99. It implies that equations could accurately describe the relations among the ε′, X_1_, X_2_, and X_3_ at the selected frequencies.

#### 2.2.2. Dielectric Loss Factor, ε′′

**Figure 4:**
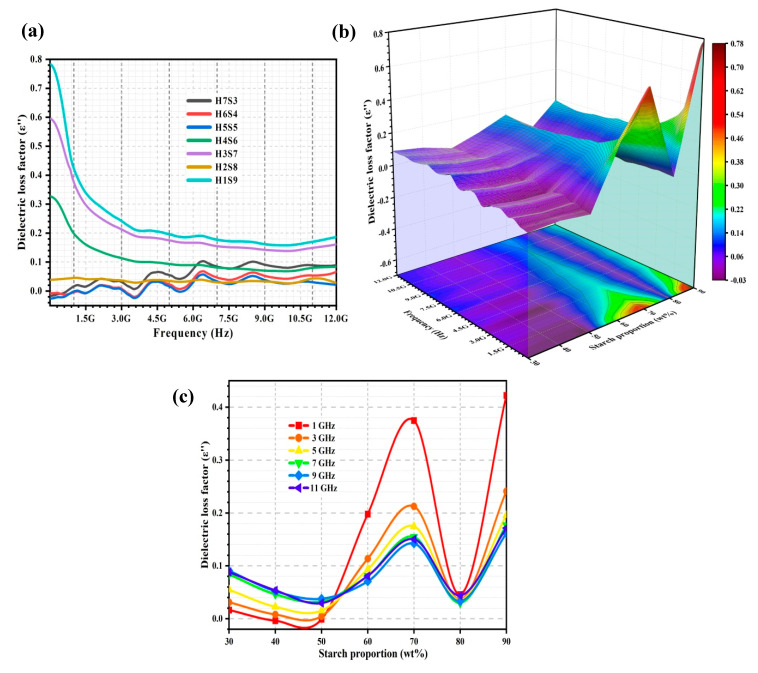
ε″ (**a**) of the porous nHA/S composites; 3D representation of ε″ of the porous nHA/S composites (**b**); ε″ curves of the porous nHA/S composites at various frequencies (**c**).

Figure 4a shows the dielectric loss factor (ε″) of the porous nHA/S composites. Figure 4b shows the 3D representation of ε″ of the porous nHA/S composites as a function of frequency (from 5 MHz to 12 GHz) and starch proportion (from 30 to 90 wt%). The variation of ε″ of the porous nHA/S composites at 1, 3, 5, 7, 9, and 11 GHz over the starch proportion can be observed in Figure 4c. The ε″ of the porous nHA/S composites are significantly influenced by the morphological features and the material proportions.

In the porous nHA/S composite with high starch proportion (60–90 wt%), the ε″ behaves more stable than the porous nHA/S composite with low starch proportion (30–50 wt%), as shown in Figure 4a. These behaviors might be due to the porous composites with high hydroxyapatite content that exceed the critical proportion. It results in the natural resonance behavior during the polarizations [27,35,51,52]. As shown in Figure 4b,c, the ε″ of the porous nHA/S composites decreases when the starch proportion increases from 30 to 50 wt%. It corresponds to the increment in the average pore size and porosity (Figure 2). Meanwhile, the decrement in the hydroxyapatite content (from 70 to 50 wt%) contributes to the decrement in the ε″ of the porous nHA/S composites. This might be due to the low hydroxyapatite nanoparticles with relatively low polar functional groups in the porous nHA/S composites. It restricts the movement of free electrons. Consequently, it reduces the conduction currents and the energy dissipation of the porous composites during the dipole polarization when it is subjected to the applied electric field [17,24,35,43].

The increment in starch proportion (from 50 to 70 wt%) leads to the drastic increment in the ε″ of the porous nHA/S composites increases rapidly, as shown in Figure 4b,c. It can be obviously seen at the low frequency. The higher starch proportion of the porous nHA/S composites induces higher intrinsic dipoles of starch and more accumulated space charges on the composite interfaces. It causes strong polarization and relative polarization relaxation processes (dissipation of energy) [7,31,45,53]. Meanwhile, the multiple scattering/reflections of the electromagnetic radiation occur frequently in the highly porous structure of the porous nHA/S composites. It facilitates the effective electromagnetic energy dissipation [39,40,52,54]. The ε″ decreases significantly as the frequency increases, as found in Figure 4a,b. It is a typical dielectric relaxation process that the dipoles/charges lag behind the phase of the applied electric field [26,43,55]. The H2S8 composite exhibits a more remarkable ε″ diminution than the other porous nHA/S composites, as shown in Figure 4b,c. This might be due to the insufficient hydroxyapatite content. This insufficiency might not contribute to the composite interfaces that encourage the space–charge accumulation during the interfacial polarization. Subsequently, it leads to the low dissipation of electromagnetic energy [24,29,52]. The highest ε″ of the porous nHA/S composite with 90 wt% starch proportion is exhibited among the other porous composites over the frequency, as shown in Figure 4a,c. The porous nHA/S composite (H1S9) with the highest starch proportion produces the dense porous structure, which also contributes to the higher homogeneity in the heterogeneous nHA/S composite that enhances the direct current conductance and energy dissipation [20,40,42].

The relationship among ε″, starch proportion, pore size, and porosity in the porous nHA/S composites can be expressed in the regression model at 1, 3, 5, 7, 9, and 11 GHz, as shown in Table 2. In the regression models, the starch proportion and porosity also have a positive effect on the ε″ of the porous nHA/S composites for all frequencies, as shown in Table 2. However, the pore size has a negative effect on the ε″ of the porous nHA/S composites over frequency (Table 2), except for the frequency of 1 GHz. The coefficient of determination (R^2^) in Table 2 exceeds 0.97, which reflects the accuracy of equations in relations between the ε″ and the parameters at a particular frequency.

#### 2.2.3. Dielectric Loss Tangent, tan δ

**Figure 5:**
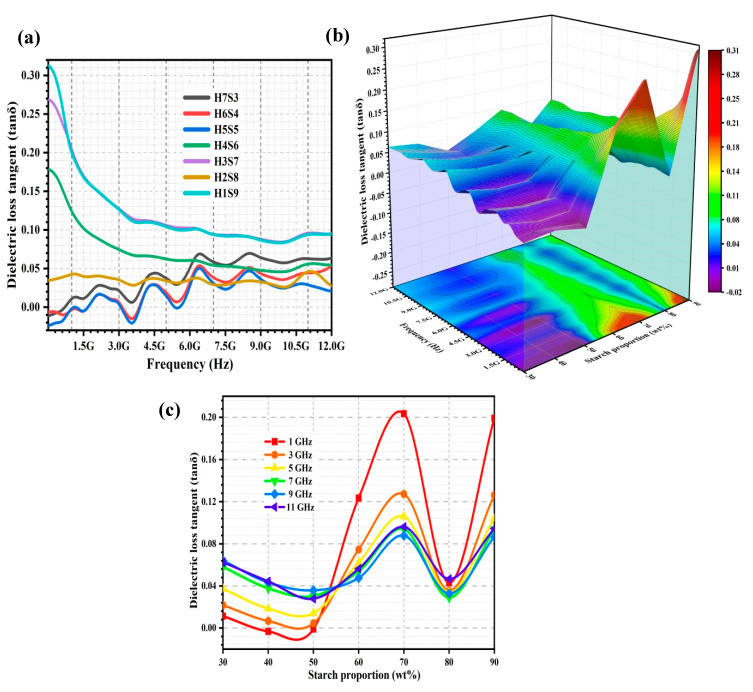
The tan δ spectra (**a**) of the porous nHA/S composites. Three-dimensional representation of tan δ values of the porous nHA/S composites (**b**). The tan δ curves of the porous nHA/S composites at varied selected frequencies (**c**).

Figure 5a shows the dielectric loss tangent (tan δ) spectra of the porous nHA/S composites. Figure 5b shows the effect of the frequency (from 5 MHz to 12 GHz) and starch proportion (range from 30 to 90 wt%) on the tan δ of the porous nHA/S composites. Figure 5c shows the variation of tan δ of the porous nHA/S composites at 1, 3, 5, 7, 9, and 11 GHz over starch proportion. It can be observed that the tan δ profile in Figure 5a is similar to the ε″ profile of the porous nHA/S composites in Figure 4a, which suggests that the tan δ is highly dependent on the ε″ of the porous nHA/S composites than the ε′.

In Figure 5a, the tan δ of the nHA/S composites with higher hydroxyapatite content, i.e., H7S3, H6S4, and H5S5, tends to increase within 5 MHz to 12 GHz, the strong dielectric resonance peaks at 2.2, 4.50, 6.4, and 8.5 GHz. The dielectric loss tangent is dependent on the conductive loss and polarization loss (dipole polarization and interfacial polarization). The dipole polarization loss increases with increasing hydroxyapatite content because of the elevated dipole concentration. The dielectric resonance peaks are contributed by the electromagnetic wave attenuation of the multiple polarization processes [21,39,48,56]. It was noted that when the starch proportion is increased from 30 to 50 wt%, the tan δ of the porous nHA/S composites decreased, as shown in Figure 5b,c. This might be due to the agglomeration effect of the hydroxyapatite nanoparticles decreased by the increment in the starch proportion in the porous nHA/S composites. The interconnected conduction pathway of the agglomerated hydroxyapatite nanoparticles is reduced, which could decrease the leakage currents and thus the conductive loss [35,45,49,57].

The tan δ of the porous nHA/S composites is similar to the behavior of ε″, where it increases when the starch proportion increases from 50 to 70 wt%, as shown in Figure 5b,c. The energy loss due to dipole polarization decreases (decrement in dipole density). Meanwhile, the energy loss due to the interfacial polarization increases (increment in composite interfaces). These energy losses occur when the hydroxyapatite content decreases. Hence, the interfacial polarization of the porous nHA/S composites with higher starch content is more dominant than the contribution of the dipole polarization. It results in a high tan δ [21,34,56]. In Figure 5b,c, the tan δ of the porous nHA/S composite with 70 wt% starch proportion is higher than the other porous nHA/S composites throughout the frequency. This might be due to the porous structures of the H3S7 composite that exhibits high average pore size and porosity. Thus, the exposed composite interface areas increase for effective electromagnetic wave attenuation [12,21,28,58]. In Figure 5b,c, it can be noticed that the porous nHA/S composite with 80 wt% starch proportion (H2S8) leads to a pronounced depression of tan δ. This is likely due to excessive starch content and significant porosity of the H2S8 composite. It creates the insulating barriers between the well-dispersed hydroxyapatite nanoparticles and therefore reduces the attenuation of electromagnetic waves [2,43,44]. In Figure 5b,c, it can be found that the tan δ of the porous nHA/S composites tends to increase as the starch proportion increases from 80 to 90 wt%. This might be due to the increment in the conductive loss, which is attributed to the relatively low average pore size and porosity of the porous nHA/S composites [15,40,50].

In Table 3, the regression model describes the tan δ as a function of starch proportion, pore size, and porosity of the porous nHA/S composites for the selected frequencies (1, 3, 5, 7, 9, and 11 GHz). Each regression model is fitted accurately with the measured tan δ of the porous nHA/S composites. It presents the coefficient of determination (R^2^) > 0.96. Hence, regression models are able to describe the tan δ of the porous nHA/S composites with a function of starch proportion, pore size, and porosity for the selected frequencies. In the regression models (Table 3), the starch proportion has a positive effect on the tan δ of the porous nHA/S composites for all selected frequencies. Similarly, the porosity has a positive effect too on the tan δ of the porous nHA/S composites for all selected frequencies, except for 1 GHz. Nevertheless, the pore size has a negative effect on the tan δ of the porous nHA/S composites for all selected frequencies (Table 3), except for 1 GHz.

#### 2.2.4. Electrical Conductivity, σ

**Figure 6:**
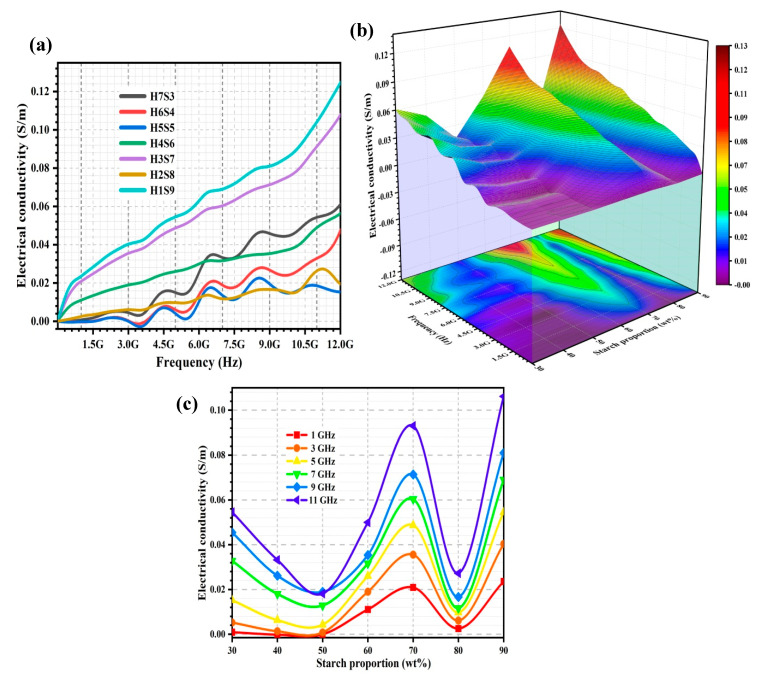
The σ spectra (**a**) of the porous nHA/S composites. Three-dimensional representation of σ of the porous nHA/S composites (**b**). The σ curves of the porous nHA/S composites at varied selected frequencies (**c**).

The electrical conductivity (σ) of the porous nHA/S composites can be deduced from the ε″. Figure 6a shows the σ spectra of the porous nHA/S composites. Figure 6b presents the variation of σ over starch proportion and frequency of the porous nHA/S composites. Meanwhile, Figure 6c shows the variation of σ of the porous nHA/S composites over starch proportion for selected frequencies (1, 3, 5, 7, 9, and 11 GHz).

As shown in Figure 6a, the σ of the porous nHA/S composites with various proportions increases significantly when the frequency increases from 5 MHz to 12 GHz, especially the H3S7 (70 wt% starch proportion) and H1S9 (90 wt% starch proportion) composites. The σ of the porous nHA/S composites increases when frequency increases. The acceleration of the movement of electrons (migrating, hopping, and tunneling) dominate the σ, which indicates compliance with the universal power law [9,42,56,59]. As the starch proportion increases from 30 to 50 wt%, the σ of the porous nHA/S composites decreases, as shown in Figure 6b,c. This might be due to the increment in the average pore size and porosity leading to the reduction in the conductive pathways in the porous nHA/S composites. Meanwhile, the number of mobile charge carriers and interparticle electron conduction distances decrease as the hydroxyapatite content of the porous nHA/S composites decreases, which restricts the electron transport mechanisms [13,16,51,60].

A drastic increment in the σ can be observed when the starch proportion increases from 50 to 70 wt% in the porous nHA/S composites, as shown in Figure 6b,c. The agglomeration effect of the hydroxyapatite nanoparticles is reduced, which contributes to the larger surface area of the nanoparticles. It can interact significantly with the starch matrices in the porous nHA/S composites and induce the formation of charge–transfer complexes. Subsequently, it forms conductive three-dimensional networks of the porous nHA/S composites that can contribute to the conduction process [10,26,45,60]. In Figure 6b,c, the porous nHA/S composite with the 80 wt% starch proportion exhibit a low σ. The starch matrix is abundantly available. These starch matrices act as the insulating barriers to suppress the tunneling current effectively between the well-dispersed hydroxyapatite nanoparticles, which results in the reduction in σ of the porous nHA/S composites [24,43,45]. Meanwhile, low hydroxyapatite content with 20 wt% and high porosity in the H2S8 composite (Figure 2) might also reduce the effective conductive pathways by inhibiting the formation of the unique conductive network structure [24,45,61]. The highest σ is exhibited at the 90 wt% starch proportion of the porous nHA/S composites, as shown in Figure 6a,c. It is because considerably small average pore size and the lowest porosity of the H1S9 composite contribute to the formation of the highly connected and denser microstructures for electron migrating and electron hopping [7,39,43,45].

The mathematical regression models of the porous nHA/S composites to express starch proportion (X_1_), pore size (X_2_), porosity (X_3_), and σ for the selected frequencies were developed, as shown in Table 4. In Table 4, the regression models show that the X_1_ and X_3_ have a positive effect on the σ of the porous nHA/S composites for all frequencies. Nevertheless, the X_2_ has a negative effect on the σ of the porous nHA/S composites for all frequencies (Table 4), except for 1 GHz. The high coefficient of determination value (R^2^ > 0.97) of the regression models (Table 4) indicates a high correlation between the σ of the studied porous nHA/S composites with X_1_, X_2_, and X_3_ at the selected frequencies.

#### 2.2.5. Penetration Depth, D_p_

**Figure 7:**
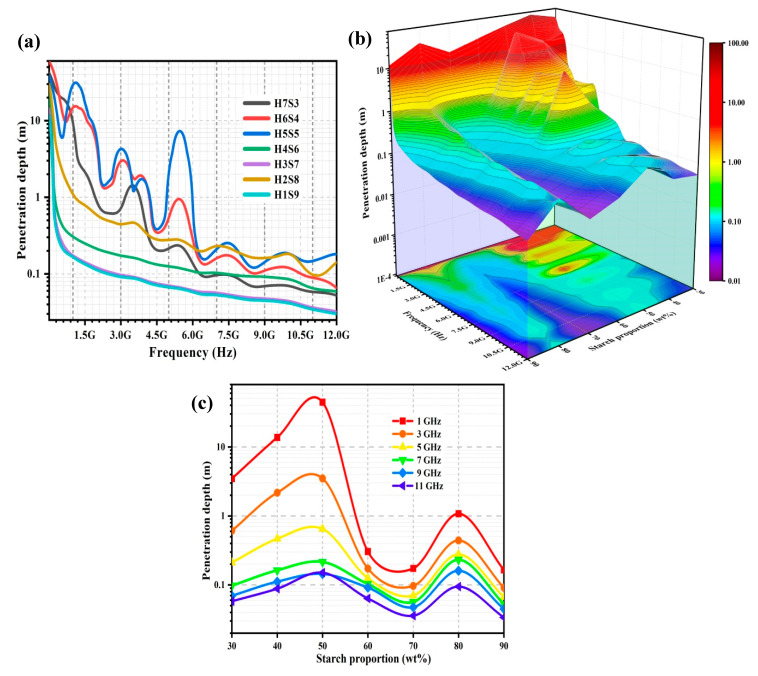
D_p_ spectra (**a**) of the porous nHA/S composites. Three-dimensional representation of D_p_ values of the porous nHA/S composites (**b**). D_p_ curves of the porous nHA/S composites at varied selected frequencies (**c**).

Penetration depth (D_p_) describes the effective depth of electromagnetic power dissipation for the porous nHA/S composites. Figure 7a shows the D_p_ spectra of the porous nHA/S composites. Figure 7b shows the effect of the frequency (range from 5 MHz to 12 GHz) and starch proportion (range from 30 to 90 wt%) on the D_p_ of the porous nHA/S composites. Figure 7c exhibits the variation of D_p_ of the porous nHA/S composites with various selected frequencies (1, 3, 5, 7, 9, and 11 GHz) over starch proportion.

In Figure 7a, the D_p_ of the porous nHA/S composites decreases when frequency increases. As the starch proportion increases from 30 to 50 wt%, the D_p_ of the porous nHA/S composites increases, as shown in Figure 7b,c. This might be due to the larger average pore size and the higher porosity of the porous nHA/S composites that contribute to the effective electromagnetic radiation penetration. The dipole polarization of the porous nHA/S composites decreases as the hydroxyapatite content decreases. This polarization leads to the low energy dissipation of electromagnetic radiation and facilitates electromagnetic wave propagation [20,21,23,62]. In Figure 7a,b, the D_p_ of the porous nHA/S composites with low starch proportion (30–50 wt%) exhibit sinuously over the frequency at low frequency. This might be due to the electromagnetic wave attenuation and resonance behaviors of the saturated hydroxyapatite content in the porous nHA/S composites when exposed to an applied electric field [21,34,40,52].

The D_p_ of the porous nHA/S composites decreases when the starch proportion increases from 50 to 70 wt%, as shown in Figure 7b,c. It is attributed to the effect of the organization of porous microstructures and enhancement of the interfacial polarization in the porous nHA/S composites. It induces the multiple reflections/scattering and efficient energy conversion of electromagnetic radiation. The low fraction of the electromagnetic wave can only be propagated through the porous composite; consequently, it causes a low D_p_ [24,28,40,63]. It can be observed that the D_p_ of the porous nHA/S composites increases as the starch proportion increases from 70 to 80 wt%, as shown in Figure 7b,c. The suppressed interfacial polarization and the highly interconnected porous structure of the H2S8 composite (80 wt% starch proportion) lead to minor energy absorption. In other words, there is a large fraction of the electromagnetic wave that penetrates through the porous composite [11,23,28,63]. As shown in Figure 7b,c, the H1S9 composite exhibits the lowest D_p_ among the other porous nHA/S composites. The highest starch proportion of the H1S9 composite (90 wt% starch proportion) exhibits the low average pore size and porosity. It restricts the propagation pathway and the D_p_ of the electromagnetic wave [23,28,39].

Table 5 lists the regression models of D_p_ for the porous nHA/S composites in the function of X_1_, X_2_, and X_3_ at the selected frequencies. The high coefficient of determination (R^2^ > 0.98) shows that the D_p_ of the porous nHA/S composites is numerically available at the selected frequencies with certain accuracy via the developed regression models. In the regression models (Table 5), the X_2_ has a positive effect on the D_p_ of the porous nHA/S composites for all selected frequencies. The X_1_ has a positive effect at low frequencies, i.e., 1, 3, and 5 GHz. Meanwhile, it has a negative effect at high selected frequencies, i.e., 7, 9, and 11 GHz of the porous nHA/S composites’ D_p_. The porosity has a negative effect on the D_p_ of the porous nHA/S composites at 1, 7, 9, and 11 GHz only, as listed in Table 5.

## 3. Materials and Methods

### 3.1. Sample Preparations

In this work, the hydroxyapatite nanoparticles (nHA) with CAS No. 1306-06-5 from Sigma-Aldrich (St. Louis, MO, USA) and the native cornstarch (S) were commercially available. The porous nHA/S composites with nHA/starch with weight percentages of 70/30 (H7S3), 60/40 (H6S4), 50/50 (H5S5), 40/60 (H4S6), 30/70 (H3S7), 20/80 (H2S8), and 10/90 (H1S9) were prepared. A starch solution (starch/water volume ratio, 1:3) was prepared by dissolving the native cornstarch in distilled water, and the pregelatinized starch solution was prepared via thermal treatment (45–65 °C) for 1 h. The hydroxyapatite nanoparticles were mixed with the pregelatinized starch solution and heated to 100 °C (10 min) for a homogeneous nHA/S mixture. The nHA/S mixture was mixed with porogen particles evenly and cast into the Teflon mold. The ratio of nHA/S mixture to porogen is 1:2, and sodium chloride particulates were used as a porogen. The nHA/S composite was dehydrated (80–90 °C) for 24 h after the composite was cooled (2–10 °C) for 2 h. The dehydrated nHA/S composite was further heated (110–140 °C) for 3 h. Then, the nHA/S composite was immersed in deionized water to leach out the porogen completely for the porous nHA/S composite. Lastly, the porous nHA/S composite was dried (85–95 °C) for 4 h after the porous composite was immersed in ethanol (95%) for approximately 5 min.

### 3.2. Sample Characterizations

The liquid displacement method (Archimedes’ principle) was used to measure the porosity of the porous nHA/S composites. The weight of samples was examined using an analytical balance, and ethanol was used as the displacement liquid. The porosity of the porous nHA/S composites was determined by using the following Equation (1):

(1) $$\mathrm{Porosity}=\frac{\left(\frac{W_{\mathrm{Wet}}-W_{\mathrm{Dry}}}{\rho_{\mathrm{ethanol}}}\right)}{\left(\frac{W_{\mathrm{Wet}}-W_{\mathrm{Submerged}}}{\rho_{\mathrm{ethanol}}}\right)}\times 100\%$$

where W_Dry_ is the weight of the dry sample, W_wet_ is the saturated weight of the sample in ethanol, W_Submerged_ is the weight of the sample submerged in ethanol, and P_ethanol_ is the density of ethanol (0.798 g/mL).

The morphological feature of the porous nHA/S composites was examined by a Hitachi TM3000 benchtop scanning electron microscope (SEM) at an acceleration voltage of 15 kV. The SEM images were obtained at a magnification of ×50. The pore size dimensions were measured using ImageJ software. Meanwhile, the dielectric properties of the porous nHA/S composites were measured by using the open-ended coaxial cable technique. The dielectric measurement was conducted by using Agilent Technologies open-ended coaxial-line probe (85070E-020) in conjunction with an Agilent Technologies E5071C network analyzer. The network analyzer and the probe must be calibrated. Air, deionized water, and shorting block were used as the calibration standards for reliable measurements. The calibration is refreshed on every measurement to increase the accuracy of the measurements. Air is used as the refreshing standard. ε′ and ε″ of the porous nHA/S composites were measured with Agilent Technologies 85070E dielectric probe kit software from 5 MHz to 12 GHz at room temperature (25 °C). ε′ and ε″ are the real component and the imaginary component of the complex permittivity, respectively, as shown in Equation (2) [18,64]:

(2) $$\varepsilon^{*}=\varepsilon^{\prime }-j\varepsilon^{\prime \prime }$$

The tan δ and σ were determined by using the following Equations (3) and (4):

(3) $$\tan\delta =\frac{\varepsilon^{\prime \prime }}{\varepsilon^{\prime }}$$

(4) $$\sigma =\omega \varepsilon_{0}\varepsilon^{\prime \prime }$$

where ω is the angular frequency (2π*f*), and ε_0_ is the free space permittivity (8.85 × 10^−12^ F/m) [16,20]. The penetration depth (D_p_) was calculated using Equation (5):

(5) $$D_{p}=\frac{c}{\omega \sqrt{2\varepsilon^{\prime }\left[\sqrt{1+{\left(\tan\delta \right)}^{2}}-1\right]}}$$

where c is the speed of light in free space (3 × 10^8^ m/s) [64,65].

## 4. Conclusions

In this study, the porous nHA/S composites were successfully fabricated using 30–90 wt% of the starch proportion. The dielectric properties of the porous composites as a function of starch proportion from 5 MHz to 12 GHz were evaluated. The porous composites with low starch proportion (30–50 wt%) exhibit high porosity due to the agglomeration effect of the hydroxyapatite nanoparticles. Then, the porous composites present low ε′, ε″, tan δ, and σ but high D_p_. By contrast, the porous composites with the high starch proportion (60–90 wt%) exhibit large average pore size due to the significant interfacial interactions between materials in porous composites, except for 80 wt%. Hence, the porous composites present high ε′, ε″, tan δ, and σ but low D_p_. Meanwhile, the porous composite with 80 wt% starch proportion presents low ε′, ε″, tan δ, and σ but high Dp. It is due to the porous structure with regular dispersion of hydroxyapatite nanoparticles, higher porosity, and relatively optimum pore size. This porous composite has a high potential for bone tissue engineering application as its robust porous microstructure is favorable for stimulating bone cell growth and tissue regeneration. In this work, it can be found that the highly interconnected hierarchical porous microstructure possesses the particular dielectric responses, which demonstrates to prove the feasibility of the dielectric characterization for bone tissue engineering application. The starch proportion, pore size, and porosity have a pronounced effect on the developed regression models (R^2^ > 0.96) for ε′, ε″, tan δ, σ, and D_p_ of the porous composites at selected frequencies. This study illustrated the relationship between microstructural features, material proportions, and dielectric behaviors of the porous composites over the 5 MHz–12 GHz frequency range. It can potentially help in the design of bone scaffolds and the development of non-destructive quality assessment equipment.

## Figures and Tables

**Table 1:** Regression equations for the dielectric constant (ε′) of the porous nHA/S composites at 1, 3, 5, 7, 9, and, 11 GHz.

Dielectric Constant Equation	R^2^
ε′_1GHz_ = −9.1691 + 0.4058X_1_ − 0.0211X_2_ + 0.1937X_3_ + 0.0671 × 10^−^^2^X_1_X_2_ − 0.7240 × 10^−^^2^X_1_X_3_	0.997
ε′_3GHz_ = −7.0670 + 0.3330X_1_ − 0.0180X_2_ + 0.1571X_3_ + 0.0550 × 10^−^^2^X_1_X_2_ − 0.5959 × 10^−^^2^X_1_X_3_	0.994
ε′_5GHz_ = −8.4368 + 0.3432X_1_ − 0.0188X_2_ + 0.1800X_3_ + 0.0552 × 10^−^^2^X_1_X_2_ − 0.6130 × 10^−^^2^X_1_X_3_	0.992
ε′_7GHz_ = −8.5438 + 0.3424X_1_ − 0.0182X_2_ + 0.1804X_3_ + 0.0536 × 10^−^^2^X_1_X_2_ − 0.6089 × 10^−^^2^X_1_X_3_	0.993
ε′_9GHz_ = −8.6129 + 0.3422X_1_ − 0.0197X_2_ + 0.1830X_3_ + 0.0556×10^−^^2^X_1_X_2_ − 0.6108 × 10^−^^2^X_1_X_3_	0.995
ε′_11GHz_ = −11.0086 + 0.3770X_1_ − 0.0193X_2_ + 0.2200X_3_ + 0.0547×10^−^^2^X_1_X_2_ − 0.6654 × 10^−^^2^X_1_X_3_	0.994

X_1_ = Starch proportion; X_2_ = Pore size; X_3_ = Porosity.

**Table 2:** Regression equations for the dielectric loss factor (ε″) of the porous nHA/S composites at 1, 3, 5, 7, 9, and 11 GHz.

Dielectric Loss Factor Equation	R^2^
ε″_1GHz_ = −3.2106 + 0.1399X_1_ + 0.0635 × 10^−2^X_2_ + 0.0494X_3_ + 0.0155 × 10^−2^X_1_X_2_ − 0.2362 × 10^−2^X_1_X_3_	0.993
ε″_3GHz_ = −1.8103 + 0.0777X_1_ − 0.1570 × 10^−2^X_2_ + 0.0306X_3_ + 0.0114 × 10^−2^X_1_X_2_ − 0.1350 × 10^−2^X_1_X_3_	0.998
ε″_5GHz_ = −1.6670 + 0.0653X_1_ − 0.2894 × 10^−2^X_2_ + 0.0306X_3_ + 0.0116 × 10^−2^X_1_X_2_ − 0.1169 × 10^−2^X_1_X_3_	0.999
ε″_7GHz_ = −2.3883 + 0.0708X_1_ − 0.4684 × 10^−2^X_2_ + 0.0447X_3_ + 0.0134 × 10^−2^X_1_X_2_ − 0.1286 × 10^−2^X_1_X_3_	0.988
ε″_9GHz_ = −2.4887 + 0.0675X_1_ − 0.5732 × 10^−2^X_2_ + 0.0477X_3_ + 0.0143 × 10^−2^X_1_X_2_ − 0.1248 × 10^−2^X_1_X_3_	0.970
ε″_11GHz_ = −2.2366 + 0.0646X_1_ − 0.7469 × 10^−2^X_2_ + 0.0459X_3_ + 0.0169 × 10^−2^X_1_X_2_ − 0.1233 × 10^−2^X_1_X_3_	0.983

X_1_ = Starch proportion; X_2_ = Pore size; X_3_ = Porosity.

**Table 3:** Regression equations for the dielectric loss tangent (tan δ) of the porous nHA/S composites at 1, 3, 5, 7, 9, and 11 GHz.

Dielectric Loss Tangent Equation	R^2^
tanδ_1GHz_ = 0.3317 + 0.0404X_1_ + 0.2313 × 10^−2^X_2_ − 0.7811 × 10^−2^X_3_ + 0.0047 × 10^−2^X_1_X_2_ − 0.0681 × 10^−2^X_1_X_3_	0.981
tanδ_3GHz_ = −0.6830 × 10^−2^ + 0.0266X_1_ − 0.0408 × 10^−2^X_2_ + 0.1030 × 10^−2^X_3_ + 0.0055 × 10^−2^X_1_X_2_ − 0.0482 × 10^−2^X_1_X_3_	0.994
tanδ_5GHz_ = −0.2094 + 0.0238X_1_ − 0.1327 × 10^−2^X_2_ + 0.5644 × 10^−2^X_3_ + 0.0059 × 10^−2^X_1_X_2_ − 0.0448 × 10^−2^X_1_X_3_	0.999
tanδ_7GHz_ = −0.9280 + 0.0312X_1_ − 0.2324 × 10^−2^X_2_ + 0.0185X_3_ + 0.0069 × 10^−2^X_1_X_2_ − 0.0583 × 10^−2^X_1_X_3_	0.992
tanδ_9GHz_ = −1.0839 + 0.0304X_1_ − 0.3070 × 10^−2^X_2_ + 0.0220X_3_ + 0.0076 × 10^−2^X_1_X_2_ − 0.0581 × 10^−2^X_1_X_3_	0.966
tanδ_11GHz_ = −0.7336 + 0.0252X_1_ − 0.4971 × 10^−2^X_2_ + 0.0189X_3_ + 0.0103 × 10^−2^X_1_X_2_ − 0.0532 × 10^−2^X_1_X_3_	0.985

X_1_ = Starch proportion; X_2_ = Pore size; X_3_ = Porosity.

**Table 4:** Regression equations for the electrical conductivity (σ) of the porous nHA/S composites at 1, 3, 5, 7, 9, and 11 GHz.

Electrical Conductivity Equation	R^2^
σ_1GHz_ = −0.1792 + 0.7807 × 10^−2^X_1_ + 0.0035 × 10^−2^X_2_ + 0.2754×10^−2^X_3_ + 0.0009 × 10^−2^X_1_X_2_ − 0.0132 × 10^−2^X_1_X_3_	0.993
σ_3GHz_ = −0.3026 + 0.0130X_1_ − 0.0262 × 10^−2^X_2_ + 0.5122 × 10^−2^X_3_ + 0.0019 × 10^−2^X_1_X_2_ − 0.0226 × 10^−2^X_1_X_3_	0.998
σ_5GHz_ = −0.4655 + 0.0182X_1_ − 0.0808 × 10^−2^X_2_ + 0.8537 × 10^−2^X_3_ + 0.0032 × 10^−2^X_1_X_2_ − 0.0326 × 10^−2^X_1_X_3_	0.999
σ_7GHz_ = −0.9318 + 0.0276X_1_ − 0.1827 × 10^−2^X_2_ + 0.0174X_3_ + 0.0052 × 10^−2^X_1_X_2_ − 0.0502 × 10^−2^X_1_X_3_	0.988
σ_9GHz_ = −1.2478 + 0.0339X_1_ − 0.2873 × 10^−2^X_2_ + 0.0239X_3_ + 0.0072 × 10^−2^X_1_X_2_ − 0.0626 × 10^−2^X_1_X_3_	0.970
σ_11GHz_ = −1.3838 + 0.0400X_1_ − 0.4621 × 10^−2^X_2_ + 0.0284X_3_ + 0.0104 × 10^−2^X_1_X_2_ − 0.0763 × 10^−2^X_1_X_3_	0.983

X_1_ = Starch proportion; X_2_ = Pore size; X_3_ = Porosity.

**Table 5:** Regression equations for the penetration depth (D_p_) of the porous nHA/S composites at 1, 3, 5, 7, 9, and 11 GHz.

Penetration Depth Equation	R^2^
D_p1GHz_ = −500.6451 + 3.4935X_1_ + 6.0880X_2_ − 0.0936X_3_ − 0.0945X_1_X_2_ + 0.0708X_1_X_3_	0.997
D_p3GHz_ = −70.8607 + 0.6315X_1_ + 0.2674X_2_ + 0.7654X_3_ − 0.4475 × 10^−2^X_1_X_2_ − 0.3925 × 10^−2^X_1_X_3_	0.981
D_p5GHz_ = −8.4976 + 0.0289X_1_ + 0.0324X_2_ + 0.0927X_3_ − 0.0619 × 10^−2^X_1_X_2_ + 0.0385 × 10^−2^X_1_X_3_	0.990
D_p7GHz_ = 1.5279 − 0.0701X_1_ + 0.6041×10^−2^X_2_ − 0.0311X_3_ − 0.0168 × 10^−2^X_1_X_2_ + 0.1343 × 10^−2^X_1_X_3_	0.999
D_p9GHz_ = 1.7830 − 0.0554X_1_ + 0.5409×10^−2^X_2_ − 0.0343X_3_ − 0.0133 × 10^−2^X_1_X_2_ + 0.1058 × 10^−2^X_1_X_3_	0.992
D_p11GHz_ = 0.5054 − 0.0270X_1_ + 0.0124X_2_ − 0.0233X_3_ − 0.0217 × 10^−2^X_1_X_2_ + 0.0717 × 10^−2^X_1_X_3_	0.995

X_1_ = Starch proportion; X_2_ = Pore size; X_3_ = Porosity.

## Data Availability

The data presented in this study are available on request from the corresponding author. The data are not publicly available due to its confidentiality.
